# User experiences with clinical social franchising: qualitative insights from providers and clients in Ghana and Kenya

**DOI:** 10.1186/s12913-015-0709-3

**Published:** 2015-02-01

**Authors:** Maia Sieverding, Christina Briegleb, Dominic Montagu

**Affiliations:** Global Health Group, University of California, San Francisco, 550 16th Street, San Francisco, CA USA

**Keywords:** Clinical social franchise, Private sector, Provider experience, Client satisfaction, Provider choice, Ghana, Kenya

## Abstract

**Background:**

Clinical social franchising is a rapidly growing delivery model in private healthcare markets in low- and middle-income countries. Despite this growth, little is known about providers’ perceptions of the benefits and challenges of social franchising or clients’ reasons for choosing franchised facilities over other healthcare options. We examine these questions in the context of three social franchise networks in Ghana and Kenya.

**Methods:**

We conducted in-depth interviews with a purposive sample of providers from the BlueStar Ghana, and Amua and Tunza networks in Kenya. We also conducted qualitative exit interviews with female clients who were leaving franchised facilities after a visit for a reproductive or child health reason. The total sample consists of 47 providers and 47 clients across the three networks.

**Results:**

Providers perceived the main benefits of participation in a social franchise network to be training opportunities and access to a consistent supply of low-cost family planning commodities; few providers mentioned branding as a benefit of participation. Although most providers said that client flows for franchised services increased after joining the network, they did not associate this with improved finances for their facility. Clients overwhelmingly cited the quality of the client-provider relationship as their main motivation for attending the franchise facility. Recognition of the franchise brand was low among clients who were exiting a franchised facility.

**Conclusions:**

The most important benefit of social franchise programs to both providers and their clients may have more to do with training on business practices, patient counseling and customer service, than with subsidies, technical input, branding or clinical support. This finding may lead to a reconsideration of how franchise programs interact with both their member clinics and the larger health-seeking communities they serve.

## Background

Clinical social franchising is a rapidly growing model for delivery of services in private healthcare markets, with 83 programs in operation or planning to launch as of 2013 [1]. Social franchises engage private providers in a contractual arrangement to provide standardized health services under a common brand name [1,2]. Participating providers are offered services such as training, branding and commodity supply, in exchange for which they are expected to provide agreed-upon services, often under certain quality conditions [3,4]. The driving hypothesis behind social franchising is that a network operating under this type of contractual arrangement can deliver improved health services in terms of access and quality [3]. At the same time, providers are expected to benefit from the technical assistance provided by the franchisor, as well as to benefit financially from branding and increased client flows [3-5].

Despite its expanding scale, recent reviews have found limited evidence for the impact of social franchising in areas including health outcomes, quality, utilization and access to family planning services [2,6]. Less attention has been given to provider and client experiences with or perceptions of franchising, factors that are also likely to influence the impact of this delivery model. The few studies that have addressed provider motivations to join or maintain membership in a franchise network have found that providers cite a number of factors, including access to medicines [7-9], social responsibility [7-9], technical improvement [7-10], improved client relationship management [11] and opportunities for networking [7,9]. There is limited evidence regarding the effect of franchising on service utilization [6]. However, one study found that franchised facilities experienced higher client volumes, suggesting that this could be a financial motivation for joining the network [4]. In Myanmar, franchised providers experienced increases in income [9], although another study on the same network found that finances were not a main motivation for joining the network and providers were concerned that their revenues might decline due to network limits on profit margins [8].

While most research on providers’ motivations to join a franchise network is limited to Asia, franchising has been found to increase client satisfaction in several contexts [4,5,11]. Quality of care and a positive provider-client relationship have been found to be important to clients’ choice to attend a franchised provider in Asian contexts [5,8] and among youth seeking family planning services in Kenya [12]. General literature on client satisfaction with private sector health facilities in Kenya and Ghana, our two countries of interest, has found that interpersonal treatment by providers [13,14], the physical environment of the facility [13,15], distance [15] and wait time [15,16] are important considerations for healthcare users.

Evidence on provider and client perspectives on social franchising in Sub-Saharan Africa, however, is particularly limited. Given that there are at least 43 networks operating in the region [1], this is an important gap in our understanding of social franchising. The objective of this study was to understand experiences with clinical social franchising, from both the provider and client perspective, in the context of three large networks affiliated with international non-governmental organizations (NGOs) in Ghana and Kenya. On the provider side, we aimed to understand perspectives on the benefits and challenges of participating in a social franchise network. From the clients’ perspective, we aimed to understand factors influencing the choice to attend a franchise facility as compared to other healthcare options.

### Setting

This study compares data across three social franchise networks: BlueStar Ghana, Tunza Kenya and Amua Kenya. Amua is the oldest of the networks, and was established by Marie Stopes Kenya in 2004 under contract from the Government of Kenya [17]. The Tunza Family Health Network and BlueStar Ghana were both established in 2008 by Population Services International Kenya and Marie Stopes Ghana, respectively [1]. As of 2013, all three networks consisted of approximately 300 providers each [1]. Amua and Tunza also have separate, but affiliated, demand creation staff [1,17,18].

All three networks are fractional franchises [17-19], meaning that the network contracts with existing private providers to franchise certain services, but those providers may also offer other services that are not supported or standardized by the franchisor [3]. At the time of their establishment, all three networks franchised only sexual and reproductive health services with a strong family planning component. The networks have since added additional services including cervical cancer screening, safe motherhood and testing for sexually transmitted infections including HIV/AIDS [1,17-19]. The largest percentage of health impact for all three networks’ services rendered, however, continues to be family planning, as measured by Disability Adjusted Life Years averted [1].

The package of services provided by the three networks is quite similar, and includes training, subsidized or free access to certain equipment for reproductive health services (e.g. intra-uterine contraceptive device insertion kits), facility branding and demand creation activities [17-19]. Both BlueStar and Tunza provide a subsidized supply of family planning commodities [18,19]. Amua franchisees are eligible to obtain some commodities free from the government of Kenya; the Amua network itself is involved in supply of products in case of government stock-outs [17]. As part of their franchise agreements, all three franchises require that franchisees attend training and adhere to quality standards [17-19]. Amua additionally stipulates that franchisees provide franchised services, and Tunza that franchisees ensure stocking of commodities and demand creation [17,18]. All three networks charge their franchisees an annual membership fee and have reporting requirements for member facilities [17-19].

## Methods

The data for this study come from semi-structured, in-depth interviews with franchised providers in Ghana and Kenya and their clients. We used in-depth interviews as the data collection method because the objective of the study was to understand providers’ and clients’ perceptions of social franchising, as well as to explore their decision-making processes related to joining the network or attending one of its facilities, respectively. We chose individual interviews over a group format to encourage respondents, and particularly providers, to be forthcoming about the disadvantages of social franchising as well as the benefits. The interviews were conducted as part of the baseline data collection for the African Health Markets for Equity (AHME) qualitative evaluation. At the time of data collection, none of the AHME interventions had yet rolled out to facilities that were existing members of the network.

### Sample selection

The three franchised networks, BlueStar Ghana and Amua and Tunza in Kenya, were selected for the study because they are the three networks in Ghana and Kenya that are participating in AHME. The national context of social franchising is quite different in the two countries; to the best of our knowledge, BlueStar is the only social franchise network currently operating in Ghana, whereas there are at least four networks in Kenya [1]. However, what all three networks from our sample have in common, in addition to the design elements noted above, is that they are affiliated with two of the world’s largest franchisors, the international NGOs Marie Stopes International and Population Services International. The most recent data available indicate that Marie Stopes International operates 18 social franchise networks globally and Population Services International operates 25, far outnumbering other parent organizations [1].

Within each network, we used a purposive sampling strategy to select facilities for inclusion in the study. Each franchise network provided the study team with lists of their existing franchised providers (i.e. those that were members of the network prior to the start of AHME) during the fieldwork preparation stage. In Ghana, we selected providers in Kumasi and Accra, the two regions in which BlueStar operated at the time of fieldwork, based on their selection for early incorporation into the AHME interventions. In Kenya, we first selected three counties – Nairobi, Machakos and Makueni – with a significant presence of both Amua and Tunza facilities, and which spanned rural and urban areas. Within each county, we selected facilities in order to ensure a mix of facility type and franchise network.

Using client volume data obtained from the franchisors, we selected a sub-set of these franchised providers as sites for in-depth interviews with clients exiting the facility. We selected the exit interview sites in order to ensure variation in geographic area and, in Kenya, across franchise networks. The selection criteria for exit interviews were that the respondent be female, between 18–49 years of age and have at least one child. In Kenya, the selection criteria were further limited to women with at least one child age five years or younger. Respondents also had to be exiting one of the selected franchised facilities after a child health, reproductive health or maternal heath visit.

### Data collection

Data were collected in Ghana in July 2013 and in Kenya in November-December 2013, in collaboration with the local offices of Innovations for Poverty Action. A team of local field staff trained by the first and second authors conducted the interviews in each country under the supervision of a local field manager. The field manager was responsible for recruiting selected facilities into the study during introductory visits prior to the start of fieldwork. Of the 27 facilities selected for interview in Ghana, four declined to participate in the study; two because of family emergencies, one because the facility was relocating outside of the BlueStar catchment area, and one because the facility had recently hired a new midwife who had not yet been trained by BlueStar. Of the 24 facilities selected in Kenya one declined to participate in the study due to lack of interest; the facility was replaced with another facility participating in the same network and located in the same county.

Field staff were responsible for recruiting clients into the study as they exited the franchise facility. In a number of facilities in both countries the team faced challenges in finding clients who met the eligibility criteria. However, we did not record the number of ineligible clients or eligible clients who refused to participate, as statistical representativeness was not an aim for this qualitative study. Female interviewers conducted all client exit interviews in both countries, whereas a mix of male and female staff conducted the provider interviews. Interviews were conducted in or just outside of the facility, in the language with which the respondent was most comfortable. As a result, interviews in Ghana were conducted primarily in Twi, with some provider interviews conducted in English. Most provider interviews in Kenya were conducted in English, with the remaining provider interviews and all client interviews conducted in Swahili.

Provider interviews covered the services offered by the facility, reasons for joining the franchise network, benefits and challenges of being part of the network, business practices, comparisons with other types of health facilities and general perceptions of provider quality. In each facility, field staff made all attempts to interview the staff member most knowledgeable about participation in the franchise network, but if that person was not available, interviewed another staff member. Most provider interviews lasted between 45 minutes and one hour. Client exit interviews covered the health reason for the visit, reason for choosing the particular facility, experience at the facility that day, satisfaction with the visit, comparisons with other types of health facilities and general perceptions of provider quality. Client interviews typically lasted 35–45 minutes. The provider and client interview guides were pre-tested and revised in each country prior to fielding.

### Data processing and analysis

Field staff recorded all interviews using digital recorders. A team of transcriptionists simultaneously transcribed and translated interviews under the supervision of a transcription manager. The transcription manager was also responsible for back-checking interviews, including ensuring translation accuracy. As the interviews were translated to preserve the original meaning, some of the resulting English is non-standard; the quotes presented below have been lightly edited for readability.

The study team coded the transcripts in Atlas.ti using an open-coding approach, in which codes and sub-codes were derived from the data rather than determined apriori. We adopted an iterative approach to developing the codebook, in which codes and sub-codes were refined over the course of the coding process as each interview was incorporated. Code families that spanned both provider and client interviews, such as provider choice, perceptions of quality and comparisons with other types of health facilities, were reconciled across the two groups of interviews. Themes were developed based on the code tree, and analyzed across country, franchise network and facility type. The analysis process indicated that data saturation was reached for the client and provider samples in both countries.

### Ethical considerations

The ethical review boards of the University of California, San Francisco, Ghana Health Services and Kenya Medical Research Institute approved the study protocol. Field staff obtained informed consent from all providers and clients prior to conducting an interview, and obtained permission from providers to conduct client exit interviews at their facility.

## Results

### Respondent characteristics

In total we interviewed 47 providers and 47 clients. Twenty-three providers were from BlueStar Ghana and 24 from Kenya; 10 from Amua and 14 from Tunza. The majority (29) of franchise facilities were health centers or clinics, followed by maternity wards (12), nearly all of which were in Ghana. The remaining facilities were hospitals (4), all in Kenya, and pharmacies (2), both of which were in Ghana. There was greater diversity in the types and scope of services offered by franchised providers in Kenya than in Ghana, where services focused more on maternity and reproductive health services. The majority of providers interviewed (31) were midwives or nurses, whereas the rest held a range of positions, from clinical officer to health assistant to doctor.

In Ghana, 21 clients were interviewed at a BlueStar facility, and in Kenya 26 clients were interviewed: seven at Amua facilities and 19 at Tunza facilities. Most clients came to the franchise facility because of a sick child (19), followed by family planning (12), a child welfare clinic (immunizations or weighing) (11) and antenatal care (5). More clients in Ghana came to the franchise facility because their child was sick, whereas more clients in Kenya came because of family planning. Over half of the clients in both Ghana and Kenya were returning clients and considered the franchise facility as their typical place of care for minor illnesses.

### Benefits of franchising and provider motivation to join the network

Across the three networks, providers saw training as the most beneficial aspect of participation in a franchise network to their practices. Providers also frequently cited training opportunities as a main motivation for joining the network at the time of recruitment. Many providers described the trainings provided by the franchise network in the areas of reproductive health and family planning as opportunities to improve or “*update*” their provision of existing services and to refresh skills they had lost due to time or lack of practice.

*Once I got the trainings, that would mean that I would be giving people what they are supposed to get. And preferably even much better than they are getting elsewhere…because mostly you will find [that] people who don’t attend trainings and seminars will always be doing things the way they used to do when they were in training maybe many, many years ago.* – Tunza provider in Makueni, Kenya

An important exception to this perception of franchise training as “*updates*” was in the area of long-term family planning provision, which a number of providers in the BlueStar and Amua networks in particular said that they had never learned prior to joining the franchise. Several providers in Ghana said that training in long-term methods and abortion services allowed them to serve clients who they would formerly have referred to another facility. Additionally, BlueStar trained nurses or midwives at the facility were now able to handle family planning clients, freeing doctors’ time for other procedures.

In addition to the clinical training they received, providers in both countries particularly valued training on client relations and counselling. Providers perceived improvements in their client approach to have had a direct impact on client satisfaction and perceptions of the quality of care offered by the facility, as much, if not more so, than changes in clinical practice.

*There had been that notion that family planning, reproductive health, a private hospital will not know [how to provide these services]…but Tunza has brought that kind of aspect of training to the staff that makes them seem friendly, and provided tools that enable you to have a dialogue with the client, so that at the end of the day the client actually makes an informed decision.* – Tunza provider in Machakos, Kenya

*When we joined Blue Star…we didn’t know certain things like counseling, or how the young nurses should approach the clients, but when we went to [training] we learned how to receive the patients.* – BlueStar provider in Kumasi, Ghana

Another widely perceived benefit of franchising, and a motivation for many providers to join the network, was a consistent supply of family planning commodities. In Ghana, providers mentioned commodity supply nearly as much as training, whereas in Kenya it was less important, particularly for Tunza providers. Providers in Ghana stressed the consistency of supply in addition to cost.

*[BlueStar] prices and Ministry of Health prices are almost the same, but we get a constant supply from them, unlike the Ministry of Health. At times you go [to the Ministry and] unexpectedly they will say we are short of this or that.* – BlueStar provider in Kumasi, Ghana

In Kenya, concerns over consistency of commodity supply through public sector outlets seemed to vary more by location, and providers discussed a broader set of benefits of franchise supply including subsidized prices, assured quality and the ease of restocking.

Other motivations to join the franchise network were more country-specific. Several providers in Ghana said that they had been motivated to join the network because they were convinced that the family planning services offered through BlueStar would be of benefit to their communities, including preventing unwanted pregnancies and unsafe abortions and saving lives. Providers in Kenya mentioned community benefit less commonly, and instead viewed the combination of training and supportive supervision offered by franchising as particularly attractive. Several providers in Kenya mentioned that as private healthcare providers they felt they had operated largely in isolation, without support or monitoring from any outside bodies, prior to joining the franchise. Few providers mentioned branding, a core component of the franchise model, as a benefit of franchising or motivation to join the network.

#### Challenges of participation in the franchise network

The challenges that providers reported with social franchising varied by network. In Kenya, there was considerable agreement among Amua providers in terms of the challenges they had with participation in the network. Most frequently, Amua providers said that the network’s reporting requirements were cumbersome and did not match with those of the Kenyan government.

*The greatest challenge is in the issue of report writing because it duplicates what we give to the government and we find that it is a lot of work.* – Amua provider in Machakos, Kenya

Several Amua providers also said that they were disappointed with the efficacy of the network’s demand creation activities, which they felt had not increased client flows as much as they had hoped. Finally, a few of the Amua providers who ran smaller facilities said that it was a challenge for them to attend trainings because they either had to find someone to staff their facility, or they had to miss trainings because they could not leave their business unattended. The challenge of attending trainings was also mentioned by a few Tunza providers, although since many of the Tunza facilities were larger, their challenge was asking for leave to attend the training rather than leaving the facility unstaffed.

Tunza providers mentioned a more varied set of challenges with participation in the network. Several providers mentioned challenges with the different aspects of payment; clients had difficulty understanding the sliding scale payment scheme used during community mobilization days, clients expected Tunza services to be free, or clients expected that Tunza would provide low-cost services beyond family planning. Other challenges only mentioned by one or two providers were that it sometimes took time to restock commodities, that the clinic was understaffed for handling client flows during community mobilization days, or that providers had hoped to be given access to more subsidized equipment.

Providers in Ghana were relatively less forthcoming about any challenges they experienced with participation in the network, although their responses were also quite varied. Challenges that were mentioned by one or two providers were that monitoring visits were too frequent and the BlueStar staff were “*always on you*”, and managing stock of family planning commodities. Another concern for providers in Ghana, which came up less frequently in Kenya, was low demand for family planning in the communities surrounding their facilities due to social or cultural reasons.

#### Impact of franchising on client flows and provider finances

The majority of providers from all three networks said that their client volumes for family planning services had increased since joining the franchise, which most attributed to the expanded services that they were able to offer after being trained. Providers in Kenya in particular mentioned increased client volume specifically in relation to the addition of long-term family planning methods to their services after franchising. A few providers also attributed increased client flows to improved quality of service.

*My clients have increased especially for family planning and cancer screening…and also the clinic is a bit outspoken [*sic*], it is known in many parts now.* – Tunza provider in Machakos, Kenya

A small number of providers in Kenya also attributed increased client flows to branding, stating that they believed clients were more confident in their services in part because of recognition of the franchise name.

*Well branding gave us a different image, a more attractive image and it’s true people know about Marie Stopes quite a lot in this locality. So because they know Marie Stopes is about family planning and such related services, we actually increased our [client] numbers and we noticed that*. – Amua provider in Machakos, Kenya

Most providers, however, did not consider client flows to be related to branding. This was particularly true of BlueStar providers in Ghana, who viewed branding as largely inconsequential, attributing increased client flows to the low cost of their family planning services, word of mouth and referrals. Yet providers also mentioned increased community awareness of the services they offered, in part due to the demand creation activities carried out by the franchise network, which providers said made potential clients more aware of the clinic’s services and helped dispel some common concerns about family planning use.

Although most providers said that their client flows had increased for franchised services, the degree to which they saw this as leading to financial benefit for the facility varied. On the one hand, a number of providers in Kenya said that because their client flows increased with the expanded franchise services, this brought more revenue into their facility.

*I never thought that it [the network] would affect our finances, but later on I realized [that] from when we entered Tunza, that is when we started getting more clients and that also means getting more money.* – Tunza provider in Nairobi, Kenya

*Long-term methods bring in a lot of money, because we get clients. Every month when we compile the reports, we see some improvements in finances generated by family planning services.* – Amua provider in Machakos

On the other hand, other providers in Kenya and nearly all of the providers in Ghana said that joining the franchise network had little or no impact on their revenues. In Ghana in particular, those few providers who thought that joining the franchise had any positive effect on the clinic finances described this as a marginal impact. Providers in Ghana more commonly said that family planning was not a high profit service, in part because the franchise kept margins low to encourage service use.

*Their drugs, depo [injectable birth control method] and the rest, are not expensive for you to say that I have injected someone and I have taken ten cedis [local currency], or I have injected someone and I have taken five cedis…The only benefit to the clinic is the increase in the number of people. But concerning profit on the drugs, it is not much.* – BlueStar provider in Kumasi, Ghana

In Kenya, several providers similarly described the provision of subsidized commodities as a means for the franchise network to share costs so that the provider did not lose money on offering family planning services.

Finally, a small number of providers in each country noted that meeting the franchisor’s quality standards increased the cost of providing services because, as one provider in Kenya said, they could not take “*short cuts*”.

*It is a bit on the higher side in terms of costs, because of this infection prevention….so there has been improvement on the side of the income, and also an increase in the expenditure.* – Amua provider in Makueni, Kenya

The increased costs of providing quality services therefore reduced to some degree the financial benefits of increased client flow for those providers who did perceive a financial impact of franchising.

#### Factors that influence client choice to seek care at the franchise facility

The large majority of clients in both countries reported that the first time they had attended the franchise facility was because a friend, family member or community member recommended the facility. Branding and client awareness of the franchise network were not mentioned as reasons for choosing to attend the franchise facility. Almost none of the clients in Ghana had heard of BlueStar, despite the fact that they were exiting a BlueStar franchised clinic. Similarly, only a few clients exiting Amua clinics in Kenya had heard of Amua, whereas slightly more clients exiting Tunza clinics had heard of Tunza, primarily through the radio, and could describe something about the brand.

*Yeah I have heard of Tunza clinics, mostly through the radio…I heard that they have good family planning services, their charges are low and they also offer good services.* – Tunza client, 27 years old, Makueni, Kenya

A small number of those clients said that radio was how they first came to know about the franchise facility. However, even in Kenya, many clients who were exiting franchised facilities said they had not heard of the franchise network name.

In both countries the main reason clients cited for why they chose to attend the franchise facility was the client-provider relationship. Clients discussed in detail how well franchise staff treated them, and described their treatment by the provider as caring, respectful and considerate. A few clients emphasized the caring nature of the franchise staff by saying they were like an “*aunty*” or “*brother*”, and referred to the franchise facility as like “*hom*e”.

*Oh they are caring. It’s like the way they treat you, you wouldn’t wait for someone to tell you to come here again. The way they get the time to take care of you…I always feel at home.* – BlueStar client, 30 years old, Accra, Ghana

*They care for people well and when you are given care, it’s just like the one you receive at home…You are given a basin, soap and tissues. There is someone who wakes you up in the morning and tells you that warm water has been brought for you to bathe…They even take you to the toilet and other places like the laboratory.* – Tunza client, 34 years old, Machakos, Kenya

Although clients in both countries emphasized the client-provider relationship, they used different language to describe how they felt franchise staff treated them differently from other providers, at times comparing this explicitly with the treatment they received at other facilities. In Ghana, clients often spoke of being “*pampered*” by franchise staff*.* They also valued not being yelled or shouted at by franchise staff, which they contrasted with their experiences at other, often public, health facilities.

*When you come here, they take care of you fine, no one shouts at you. That is why I bring him [child] here…There are some places when you go and you don’t know the direction and you ask the nurses, then they will be angry and they will be shouting at you.* - BlueStar client, 28 years old, Accra, Ghana

In Kenya, clients emphasized how staff were considerate of their time and ensured that clients were attended to quickly, which they also contrasted with their experiences of long queues and staff shortages at public facilities.

*I love it here [*sic*]. There is no queue…Maybe there is just one person ahead of you or maybe two, but the speed that they serve you with, they serve you quickly and then you go. You don’t wait. But at [government hospital], the bench awaits you!* – Amua client, 31 years old, Nairobi, Kenya

Another reason clients in both countries gave for choosing the franchise facility was past experience of getting better after attending the facility. Clients described having confidence in the franchise staff’s ability to help them or their children get better. They valued that franchise staff conducted tests and procedures, and prescribed quality and effective medicine, which was especially important to clients when it came to their sick children.

*They treat them [children] to get well, but if you go to another hospital and you leave, when they give you medicines you still don’t get well. The sickness remains the same unless you take him somewhere else, so me I always come here.* – BlueStar client, age unknown, Accra, Ghana

*It’s just because whenever I bring him [child] here, he is treated and he gets well. It’s not like when I take him to other places where he develops other problems and continues crying into the night. He doesn’t develop those problems whenever I bring him here.* – Tunza client, 27 years old, Makueni, Kenya

A number of clients in Ghana in particular also mentioned continuity of care as a motivation for attending the franchise facility. Clients appreciated having a continuing relationship with franchise staff and being familiar with the facility; a number of these clients had attended the franchise facility for antenatal care, delivery or postnatal care, and preferred to continue care at the franchise facility for their children.

*When I was pregnant, this hospital was the place I was coming. I delivered here and so if something is wrong with him [the child], they would know much. They will know everything about it.* – BlueStar client, 30 years old, Accra, Ghana

### Client satisfaction

Clients reported high levels of satisfaction with their visit to the franchised provider for a range of reasons that were closely related to their reasons for choosing to seek care at the facility. These reasons included the perceived quality of medical care received, how polite, friendly and caring the providers and other staff were, short waiting times and facility cleanliness. A number of clients highlighted aspects of the process of receiving care as important factors in their experience. For instance, as a result of the positive relationship clients had with franchise staff, a few clients said that they felt comfortable discussing problems and asking questions of the provider.

*I have been to many hospitals…. [here] they are sociable and when you come and something baffles your mind [*i.e. *you are confused], [she] lets me go off topic.* – BlueStar client, 38 years old, Kumasi, Ghana

Clients contrasted this sense of feeling “*free*” to talk with franchise staff with experiences of being yelled at, criticized or treated harshly at other facilities for asking questions or doing things that providers perceived as wrong. One client, for example, highlighted this issue with regards to her concerns over her baby losing weight:

*Maybe if you get one [health staff] that is harsh, you fear. His approach only makes you fear to ask him the reason. The baby is just weighed and then you get out and go. But because I am free with them [franchise staff], I asked and he told me [why].* – Amua client, 31 years old, Nairboi, Kenya

Although the majority of clients did not mention involvement in decision-making, it did seem to be an aspect of engagement and satisfaction for a few respondents. One client in Kenya described her appreciation of being involved in the decision-making process with regards to choosing a family planning method, and not just being told what to do by the provider.

*I am told about the advantages and disadvantages [of family planning] before I am told to choose. There are some clinics where you are just ordered on what to do.* – Amua client, 36 years old, Makueni, Kenya

### Reasons why clients may attend another health facility

Although clients expressed high levels of satisfaction with the franchise facility, in both countries they had a sense of the limits to the scope of services franchised facilities could offer. Correspondingly, clients gave a number of reasons for why they might choose to attend another health facility, and particularly public sector hospitals, which included a wider range of services (laboratories, surgery), the ability to handle serious illness or critical conditions, and the presence of specialists for less common conditions. Despite numerous complaints with the functioning of public health facilities, a number of clients in both countries still considered public facilities to be the most comprehensive and surest source of care available to them. Cost was also a consideration mentioned by a small number of clients, particularly in Kenya, who said that services at the franchise facility were more expensive than those offered by the public sector.

*You know we have already talked about money matters…Now in case I have any other problem, I will just bring him [baby] here if I have money. And if I don’t have money, it will force me to run to [government hospital].* – Amua client, 31 years old, Nairobi, Kenya

## Discussion

According to franchising theory, the membership benefits of a social franchise include trainings, supply and subsidy of commodities, and the business and reputational enhancement that comes from association with a known and well-marketed brand [20]. Improved reputation is in turn expected to increase client numbers for both franchised and non-franchised services, ultimately leading to increased income for franchisees [3]. Findings from in-depth interviews with providers of three social franchise programs in Ghana and Kenya indicate that training opportunities and commodity supply are overwhelming the two most commonly perceived benefits of participation in a social franchise network. These two aspects of social franchising also appear to be key motivations for providers to join a network. We find other, more minor perceived benefits of social franchising to be more country-specific, which in Ghana included community benefit and in Kenya integration into a support network.

Overall, however, the perceived long-term value of social franchising in terms of business sustainability was low among participating providers in these two contexts. This is not to say that providers did not perceive any specific improvements in their facility or practices after joining the franchise, but that they did not credit those improvements (with the exception of client care) with having a broad impact on the facility’s operations. In a critical example for franchising theory, although providers thought that client flows increased for franchised services – in these contexts, family planning services – many did not associate this with financial benefit to their facility. This may be in part due to the fact that all of the networks studied were fractional, and that the percentage of providers’ business that came from family planning appeared to vary widely. Nevertheless, the lack of attribution of benefit to franchising, whether or not the facilities were in fact experiencing changes, reflects an important gap in how franchise networks are demonstrating the long-term impact of their activities in a manner that is understandable and valuable to their members.

Although branding is considered to be a key element of social franchising’s value to providers, it is notable that the majority of providers in this study did not ascribe much impact to branding. This was particularly true in Ghana, where none of the providers thought that branding affected client flows or perceptions. The lack of value providers attached to branding was consistent with the fact that the large majority of clients exiting franchise clinics did not recognize the name of the franchise network. From both the provider and client perspectives, word of mouth and personal referrals were more important factors drawing clients to the facility than recognition of the franchise brand.

There was also considerable agreement among providers and clients regarding the importance of the client-provider relationship to positive perceptions of the franchised facility. Providers valued the training they received on client relations; changes to their organizational or consultation practice, prompted by these trainings, were a theme that was as important as changes to clinical practice, if not more so. Clients in turn overwhelmingly cited a positive client-provider relationship, including the caring and respectful nature of staff and prompt service, as their motivation for attending the franchise facility. Together, improvements to the client-provider relationship was one of the main benefits of social franchising from the perspective of both providers and their clients.

It is possible that the providers’ focus on the client relationship, and therefore the positive dynamic that led clients to return to the facility, pre-dated the provider joining the franchise network. In order words, if training in client care is what attracted providers to join the network, franchised facilities will be positively selected for attentiveness to clients even without the franchise training. However, the fact that there were a variety of factors, including training on other topics, that motivated providers to join the network, indicates that this is certainly not the only selection factor. Whether interest in client care was created by the franchise or is a reflection of the franchise responding to its’ members interests, is not an issue that has received much attention in franchising theory.

Despite these broad similarities, differences did exist in the perspectives of clients and providers across the two countries and three networks that suggest that the value of franchising differs across contexts and network structures. The challenges that providers reported with their participation in a franchise were largely network-specific or, in the case of Ghana, related to sociocultural issues regarding the services the network was franchising. Providers’ valuation of certain components of the franchise package, particularly branding, also varied by location and network. It was unclear whether these valuations varied based on providers’ priorities in a given context, or differences in the way the networks operate on the ground, including recruiting facilities into the network and investing in aspects such as branding, although it seems likely to be a combination of both.

There was also considerable variation in the sizes and services of the franchised facilities, particularly in Kenya, and the relative importance of commodity supply to different providers appeared to be affected by how easy it was for them to access commodities elsewhere. In addition to influencing facilities’ priorities, such factors suggest that the position of a facility within its local health system will affect the acceptability as well as the perceived value of franchising. For instance, the greater diversity of the facilities in Kenya in terms of size and scope of services may be reflective of the local markets in which they compete, and may also be one reason for the somewhat greater value placed on branding in Kenya. Market competition may also explain Kenyan providers’ interest in the support system and networking opportunities provided by the franchise networks. While some such variation in size and scope is to be expected in networks as large as the ones in this study, this issue again points to the need for a critical review of the meaning and importance of standardization in the context of fractional franchise networks that work with previously existing facilities.

### Limitations

Several limitations of this study should be kept in mind. As a qualitative study based on purposive rather than random sampling, our results are not generalizable to all of the franchised facilities in the three networks studied, or to other social franchise networks. The franchise networks included in this study are all fractional franchises operated by two, large international NGOs. Although these NGOs represent a substantial portion of social franchises globally, the experiences of providers participating in their networks likely vary across countries, and are different from those of providers who are members of smaller or full franchise networks. In particular, there are other social franchises in Kenya that are operated by local NGOs [1], and the findings of this study are not generalizable to those networks.

There is likely a degree of courtesy bias in both providers’ and clients’ assessments of the social franchise, which may be one reason for the reluctance of some providers to discuss challenges with their participation in the network. We also did not verify providers’ statements about the impact of franchising on their profits and client loads against their clinic records; our findings are rather based on providers’ perceptions of how franchising has impacted their facility finances. Despite these limitations, this study is one of the few to examine provider and client perceptions of social franchising together, as well as one of the few studies to address social franchising in Sub-Saharan Africa.

## Conclusions

The results of this study support evidence from several other settings that has shown that the longer-term benefits of franchising are often elusive, and even when they do accrue, providers are more focused on the immediate, proximate, benefits of membership [7-9]. Training and subsidies are highly rated by providers not because they are more valued than other benefits, but because other benefits are often so difficult to discern [c.f. 7–9]. Findings from this study confirm the high value of immediate franchising benefits to providers, but add a new, and important, insight: the educational opportunities that providers value most are not necessarily clinical, and include business and customer relations training, which providers view as new and important.

The importance of these non-clinical skills can be understood by what our study learned from clients of social franchises. Echoing franchise clients in Nepal, Pakistan, India and Myanmar [4,5,7], the clients in our study emphasized the importance of provider attitude, attentiveness and engagement. These provider characteristics are precisely the measures of quality that other studies have shown to matter most to clients seeking outpatient care [21-23]. The agreement between the value providers place on business and service-oriented training, and the value clients place on receiving attentive care, offers a new way to understand both the real and potential value that social franchise programs offer to their members and the populations that are their ultimate beneficiaries. However, our findings suggest that there is a need for new approaches to communicating that value to providers and clients, and linking perceptions of value to the services they receive through franchising. In addition, larger studies that can quantify the impact of franchising in terms health impact, clinical quality and client flows, in addition to client satisfaction, are needed in order to better understand and assess the return to the substantial investments currently being made in this delivery model in Sub-Saharan Africa and elsewhere.

## Abbreviations

AHME: African Health Markets for Equity
NGOs: Non-Governmental Organizations
